# A Systematic Approach to the Application of Soft Tissue Histopathology in Paleopathology

**DOI:** 10.1155/2015/631465

**Published:** 2015-08-06

**Authors:** Christina Grove, Oliver Peschel, Andreas G. Nerlich

**Affiliations:** ^1^Institute of Legal Medicine, Ludwig-Maximilians University of Munich, 80336 Munich, Germany; ^2^Division of Paleopathology, Institute of Pathology, Academic Clinic Munich-Bogenhausen and Munich-Schwabing, 81925 Munich, Germany

## Abstract

The application of histology to soft tissue remains offers an important technique to obtain diagnostically important information on various physiological and pathological conditions in paleopathology. In a series of 29 cases with mummified tissue ranging between 16 months and c. 5.200 years of postmortem time interval, we systematically investigated paleohistology and the preservation of various tissues. We established a reproducible histological ranking system for the evaluation of mummified tissue preservation. The application of this scheme to the series showed good tissue preservation of tissues with high connective tissue content but also fat tissue and connective tissue rich organs, such as lung tissue, while most other internal organs were less well preserved despite highly different postmortem time intervals. There are some organs with only poor conservation even in short term periods such as the kidneys and CNS. Artificial mummification does not provide better conservation than naturally mummified tissues; “cold” mummies may be much better conserved than those from desert areas. The identification of specific pathologies underlines the potential power of paleohistology.

## 1. Introduction

“Some human diseases can be diagnosed from ancient skeletal tissue, but a much greater number can be discovered by examination of preserved soft tissues.” This statement by Aufderheide [1] from 2000 still holds very true and reflects a paradigm of modern paleopathology. Although used since very long times in paleopathology, soft tissue histopathology has only been applied in few instances. Nevertheless, some of the very first paleopathologic mummy studies provided conclusive results due to histopathology, for example, in the diagnosis of schistosomiasis [2]. The subsequent extensive “mummy projects,” for example, the Manchester mummy project [3] or the Philadelphia University mummy project [4], also included histology of soft tissue. Thereby, important data could be retrieved.

Besides the limited source of soft tissue remnants, the lack of a systematic investigation of the application of histology seems to be a limiting factor for the use of soft tissue histology. In the available literature as yet no such study can be found. In the present study, we therefore performed a dual investigation, with one part covering a systematic investigation on human tissue samples from exhumations of more recent origin and a second part on human mummified tissue of various historic sources covering a wide range of time periods. Although especially the second part is strongly limited by the available tissue (which in turn depends on the accessibility of the tissue of a mummy), this study offers insight into the conservation of tissues under certain “storage” conditions. In summary, our study provides evidence that soft tissue histopathology is applicable to various organ pathologies and that the tissue conservation generally differs between certain organs.

## 2. Material and Methods

### 2.1. Material and Samples

The study was conducted on a total of 29 mummies covering a range of time between death and investigation between 1.25 years (yrs.) and c. 5.200 yrs. All cases were selected due to their good to excellent macroscopic state of conservation.

The study was initially divided into two groups: (i) the first group consisted of 17 complete/near complete mummies of the 19th till the 21st century where we had access to the whole body and were able to perform typical autopsies. (ii) The remaining 12 “cases” were significantly “older” and came from historic individuals of various places and time periods where we had mostly access only to limited material, mostly due to restrictions in the accessibility and in order to remain as little destructive as possible. In 4 of group II specimens, however, several organs were available for investigation, close to the material of group I.

**Figure 1:**
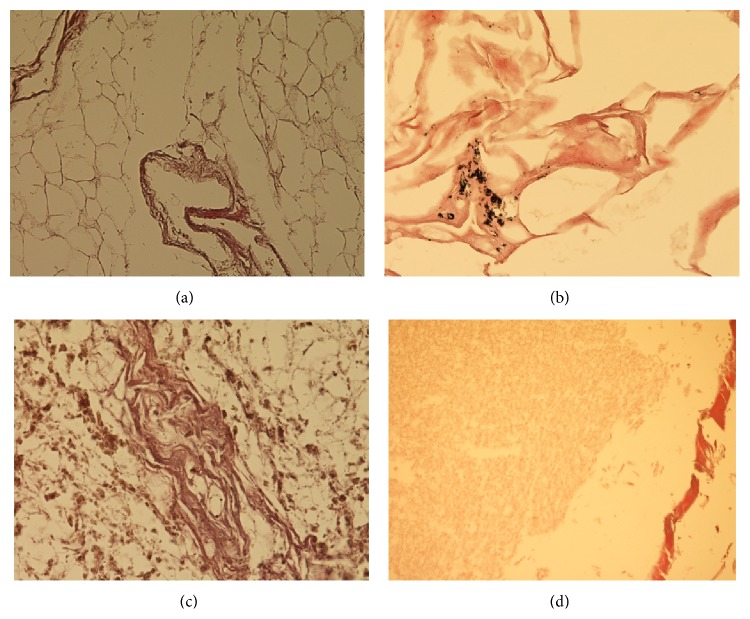
Histological features of mummified tissue from individuals with a postmortem time interval between 16 months and c. 5 yrs. (a) Fat and connective tissue (subcutis) from mummy 3 (17 months) showing good conservation of tissue structures despite the loss of cell nuclei. Note the similarly well-preserved small blood vessel. (b) Lung tissue from mummy 7 (18 months). The alveoli are distended, and in the interstitium small deposits of anthracosis pigment prove the pulmonary origin. (c) Liver tissue from mummy 12 (5 yrs.) showing severe postmortem diagenesis of the hepatocytes but retained tissue structure with small portal fields. Note the pigment that proved to be the results of postmortem oxidation products. (d) CNS tissue from mummy 12 (5 yrs.). The structure of the parenchyma has gone lost; on the right side a small sheath of connective tissue indicates the meninges (all examples: original magnification ×200, staining: H&E).

**Figure 2:**
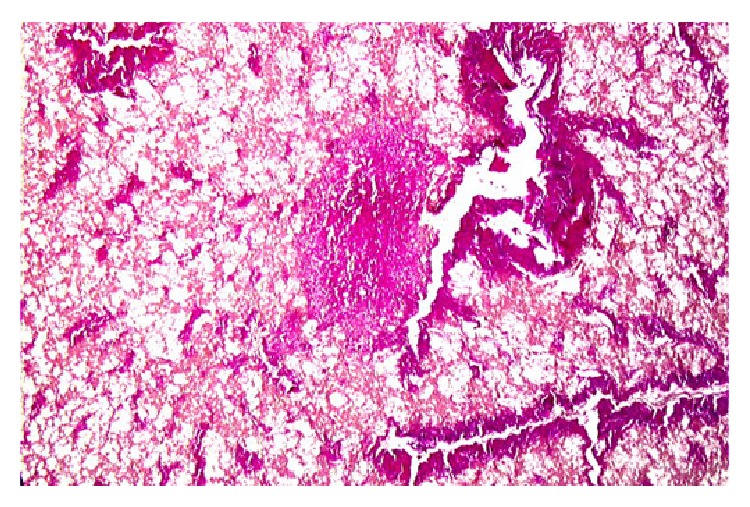
Liver tissue from the (natural) crypt mummy 12 (36 yrs.). Despite significant diagnosis here again the liver structure is retained by the presence of portal fields. In the center a nodular formation indicates the remnants of a tumor nodule (diagnosis: metastatic carcinoma) (original magnification ×100, H&E).

*Group I*. Twelve of the most recent cases came from the files of the Department of Legal Medicine of the Ludwig Maximilians University of Munich that had been obtained after exhumation for medicolegal reasons between 16 months and 5 years after death with more or less intense natural mummification. Ten cases (with a postmortem time interval between 16 and 19 months) had been autopsied from one cemetery site in Southern Bavaria (Sonthofen) as a result of a homicide series investigation (Figure 1). Unfortunately, in those cases no skin tissue was retained since this seemed to be not necessary for the forensic examination. Surprisingly, all these cases still presented with extensively preserved soft tissues including internal organs. Two more cases had been autopsied also for medicolegal reasons. One had been found as a naturally mummified corpse 5 years after death in her living apartment; the second case was a crypt burial (Figure 2).

**Figure 3:**
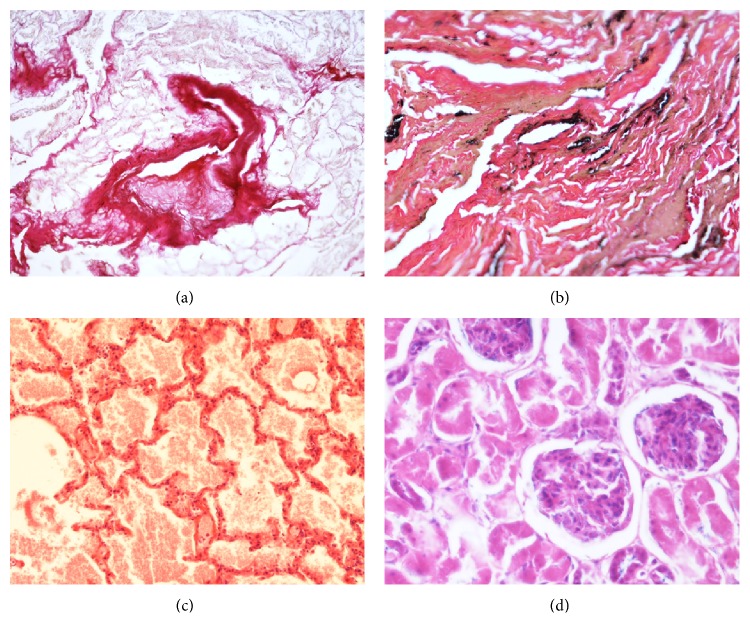
(a) A natural mummy from a German crypt of AD 1851 (14, 163 yrs.). The soft tissue is in a very well conservation status with fat cell residues and a small arteriolar blood vessel (center). (b) Lung tissue from mummy 14 with collapse of the air spaces, abundant anthracosis pigment, and small residues of a proteinaceous exudate (obviously remnants of a lung edema). (c) The artificially mummified infant 17 (198 yrs.) shows extremely well-preserved tissue structures of the lung, again with extensive residues of a (terminal) pulmonary edema. Here, the artificial conservation even has retained cell structures! (d) A section of kidney tissue from mummy 17 again with excellently preserved tissue structures as visible from glomeruli and residues of tubuli (original magnification ×200; ((a)–(d)) H&E).

In addition, we were able to investigate a small series of historic mummies of mostly natural mummification processes (except for one child mummy, see below). In this series the mummies came from a South German crypt dating to the post-Napoleonic period (AD 1841–1859) (Figure 3); one artificially mummified infant of that crypt had died in 1816 AD and had been prepared as an artificial mummy. This is described more in detail below.

**Figure 4:**
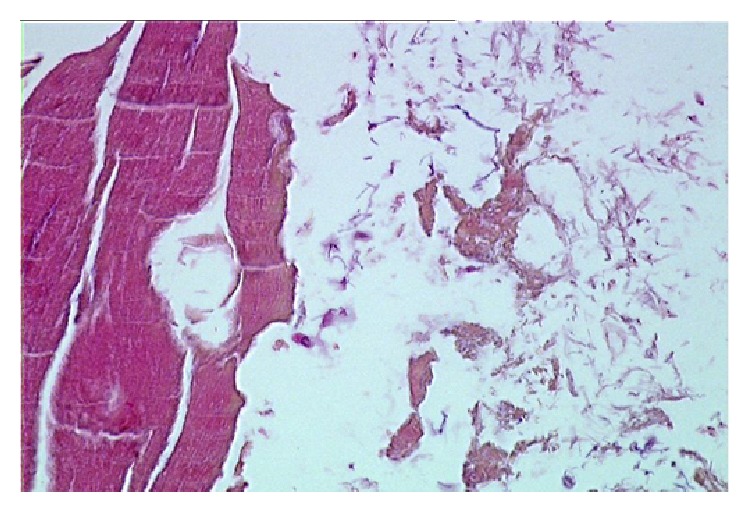
Soft tissue from the mummy skull 21 (c. 1.650 yrs.) showing a broad connective tissue plate (left) and fat tissue (right) with central extensions of a fresh perimortal bleeding (original magnification ×200, H&E).

**Figure 5:**
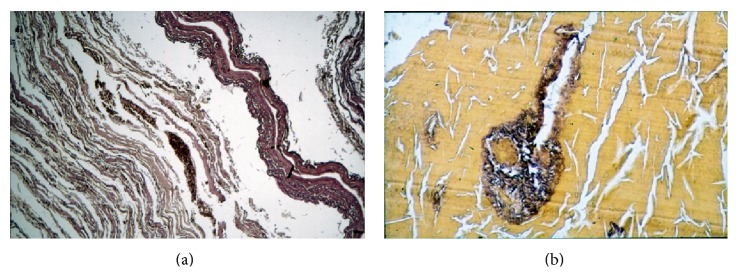
Ancient Egyptian mummy 23 (c. 2.900 yrs.) soft tissue histology. (a) Lung tissue sample showing a major blood vessel (upper part) and collapsed alveoli (lower part). Between the alveoli small deposits are seen that proved to be older bleeding residues due to a parasitic pulmonary infection. (b) Liver tissue from mummy 23 with excellently preserved portal fields (center) and the residues of the surrounding parenchyma (original magnification: (a) ×100, (b) ×200; (a) H&E; (b) van Gieson connective tissue stain).

**Figure 6:**
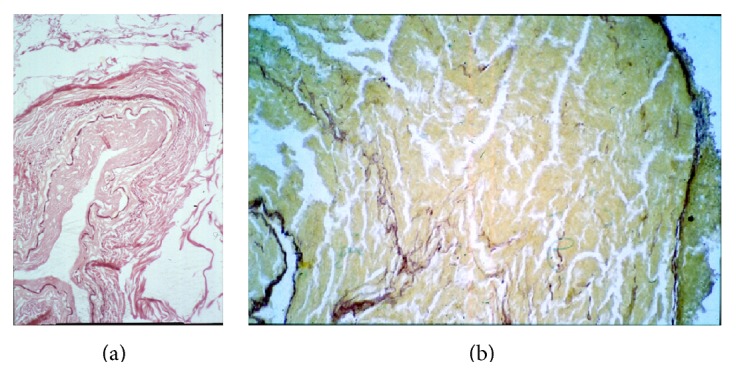
Ancient Egyptian mummy tissue from case 24 (c. 2.900 yrs.). (a) A small coronary artery shows significant intimal arteriosclerotic thickening. Note the excellently preserved internal elastic lamina. (b) Myocardial tissue with tiny connective tissue sheaths between the residues of the cardiomyocytes (original magnification: ×200; Elastica-van Gieson stain).

**Figure 7:**
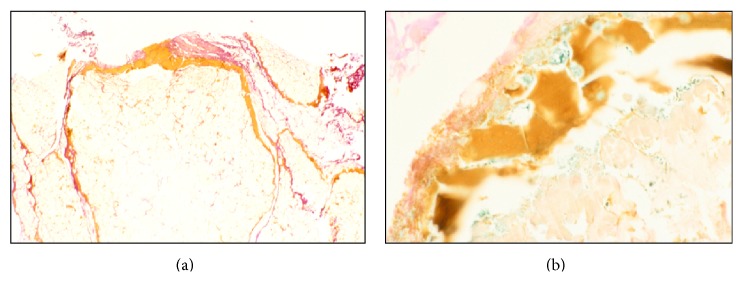
Subcutaneous tissue from the Iceman “Ötzi.” (a) The well-preserved fat tissue is separated by a fresh bleeding zone. (b) More on detail, the bleeding zone shows on special staining (Prussian blue) the remnants of haemosiderin-positive macrophages (blue) indicating a survival period of several days ante mortem (original magnification: (a) ×150; (b) ×300; (a): H&E; (b): Prussian blue stain).

*Group II*. These “cases” were significantly older than that of group I; however, in most cases the available samples were much more restricted due to conservatory reasons of the objects. From one young female mummy from South America, dating into the Nazca period between 1451 and 1642 AD [5], soft tissue samples were obtained from skin and the rectal wall. Additionally, three naturally mummified skulls from South American individuals were included dating to c. 350–500 AD [6] (Figure 4). Furthermore 7 human mummies came from ancient Egypt (c. 1900–700 BC) [7, 8] (Figures 5 and 6) and several soft tissue samples were available for this study from the Neolithic glacier mummy “Ötzi” (c. 3.200 BC) [9, 10] (Figure 7). In four of the ancient Egyptian cases we had been able to perform more extensive analyses, since several internal organs had been preserved as organ packages within the body cavities or in adjacent Canopic jars providing access to comparable tissue types than in group I (Figures 5 and 6). Detailed information on the material and the underlying cases is summarized in Table 1.

The material had been obtained from autopsies or small local tissue excisions and covers in most cases (at least in group I) several organs from different regions of the body that had been identified by macroscopy. In some cases (group II), only isolated organs could be identified by macroscopy due to their position within the body and/or their morphology, while other internal organs obviously had disappeared or were not accessible without major destruction of the mummy. In the South American mummy skulls [6] and in the Neolithic Iceman [9, 10], only small samples from skin and subcutaneous soft tissues were available, but with no internal organs.

### 2.2. Tissue Processing

In order to be able to perform histology, the dry and brittle mummy tissue has to be rehydrated. This procedure is highly critical for the further processing of the material and seems to be crucial for the tissue preservation. The samples were generally prepared according to the technique described by Ruffer in 1909 [11] with slight modifications. Briefly, the samples were immerged into a solution composed of 2–4% formaldehyde solution, pH 7.4, supplemented with 5% sodium carbonate. The sample was gently shaken for 12–16 hours in order to ensure optimal penetration of the solution into the material. After this time period the solution was replaced by 4–6% formaldehyde, pH 7.4 without any further addition for another 12–16 hours (postfixation).

The rehydration procedure is very critical for the result. In general, only in a part of cases a successful rehydration is possible. As yet, there exists no indicator that may help to discriminate between both possibilities.

The organs of the artificial infant mummy (“Wack-1”) had not to be rehydrated, since those internal organs had been stored for 200 years in ethanol. In this case, however, skin and soft tissues had to be rehydrated and these were treated as in the other specimens. The samples of the Neolithic cold mummy Ötzi were treated as those of the other specimens.

Following rehydration and fixation, mostly the fixation fluid had turned (dark) brown indicating that the solution contained oxidation products that have been removed from the tissue. Poorly conserved tissue may completely be dissolve and “disappear.” Subsequently, the now rehydrated material is further processed as routinely done in a modern histopathology laboratory. It is finally embedded into paraffin wax.

From the paraffin blocks thin sections of 2-3 *μ*m thickness are cut and stained with various routine stainings, including haematoxylin and eosin (HE), van Gieson's connective tissue stain, PAS-stain, Prussian blue staining, and eventually other special stains if required. All detailed staining procedures have previously been described in detail [7, 8].

### 2.3. Tissue Evaluation

All sections were evaluated by light microscopy as routinely performed. In order to render the observations better comparable, we applied a semiquantitative scoring system with a series of criteria that may help to define the status of the embedded tissue.

These criteria are presented in detail in Table 2 and comprise identification of tissue integrity, presence of characteristic tissue structures or substructures (e.g., cartilage of the bronchial tree in lung samples; typical layering of elastic membranes in arterial/arteriolar blood vessels), presence and extent of artefacts from preprocessing storage/decay, and staining properties in various routine stainings. In order to render this scoring system less subjective, we defined for each criterium distinct qualitative and whenever possible quantitative aspects that should enable a proper classification. The criteria are described in detail in Table 2. For further evaluation, these criteria were first tested in a subset of 10 tissue sections of the “modern” samples (group I); since this evaluation revealed good applicability, those were applied to all cases in a systematic manner. Finally, the resulting points were added to a specific summation score which was used to classify the conservation status of a sample/organ (Table 3). Generally, this indicates the histologic conservation status into 5 grades (grades 0 to 4, with 0 being the worst and 4 being the best status).

Besides this (semi)quantitative evaluation of the diagnostic features of all tissues/organs present, we recorded pathological findings in a qualitative way.

## 3. Results

### 3.1. Rate of Successful Analyses

All 29 cases included in this study provided tissue preservation sufficient for evaluation. We had no case with complete loss of tissue during the rehydration procedure. This may clearly be due to a “preselection” of the cases, since we had selected only those cases with good to excellent macroscopic soft tissue conservation. Furthermore, we chose the most “ancient” technique of rehydration which proved, at least in our hands, to be the most successful when compared to other techniques [12–15].

### 3.2. Diagnostic Evaluation Scheme

The evaluation scheme applied in this study was tested first for its applicability for the evaluation of the material. The resulting points could be clearly transferred into the ranking system. Thereby, every tissue/organ under investigation could unambiguously be characterized and classified.

The application of the system was first tested by two independent researchers (Christina Grove and Andreas G. Nerlich) showing a high rate of concordance and low interobserver variability. This was evaluated using percent agreement and kappa statistics. According to Landis and Koch [16], the agreement was rated as follows: Kappa between 0 and 0.2 indicates slight agreement, between 0.21 and 0.4 fair agreement, between 0.41 and 0.60 moderate agreement, between 0.61 and 0.8 substantial agreement, and from 0.81 excellent agreement. Absolute agreement would be 1.0. Frequency of disagreement was calculated for each sample. In the present assessment the percent agreement ranged at 88,9% for all evaluated samples; the weighted kappa value was at 0.81 indicating good to excellent agreement.

Finally, the score values were statistically evaluated between the “cases” from groups I and II with a standard correlation test (with *p* < 0.5 being regarded as statistically significantly different).

### 3.3. Observations in Naturally Mummified versus Artificially Mummified Tissues as well as in “Cold Mummy” Tissues

Although the number of cases with artificial mummification is significantly lower than the naturally mummified ones in our series, the direct comparison shows no statistical difference in the values of tissue conservations between both (*p* = 0.8). The only exception is the young infant that had been artificially conserved with an excellent preservation of almost all tissue structures. In this young infant (“Wack.1”), dying at AD 1816 at the age of approximately 1 year, all internal organs had been kept in ethanol until present time, while the body obviously had been dried by external application (and internal treatment of the body cavities) by a mixture of sodium-/potassium- and magnesium-sulphate salts. Despite minor differences in conservation of internal organs and skin/soft tissues, all specimens in this case showed excellent conservation (Figures 3(c) and 3(d)). Only the extent of some artefacts was higher in the dried tissue than in the fixed material. Accordingly, skin and the subcutaneous fat and musculature were also excellently preserved.

The exceptionally old tissue samples from the Alpine iceman (Ötzi) were also very well preserved allowing the identification of various substructures of the tissue without difficulties. Here, unfortunately, we had no access to internal organ samples that were not available at the time when our study had been granted the authorities. A direct comparison between the conservation status of Ötzi's skin and subcutaneous tissue structures with significantly “younger” ones revealed a significantly better preservation of the soft tissue in this “cold” mummy tissue.

### 3.4. Tissue Preservation with respect to the Postmortem Time Interval

In the next step we evaluated the tissue preservation with increasing postmortem time interval, especially with group I samples, and also in comparison to group II material. A (slightly) statistically better degree of preservation is seen for blood vessels (*p* < 0.5) and adipose tissue (*p* < 0.5), while the other tissues did not reveal a statistical degree of difference at all (skin and connective tissue) or obviously due to the few tissue samples available in group II (lung, heart, liver, kidney, and CNS). However, the values for the kidneys and the CNS samples were very low in both groups. Thereby, we did not observe a general or even gradual loss of tissue structures with increasing postmortem time interval, at least in our series that covered the postmortem time interval between several months and several centuries/millennia.

In addition, in our series we detected differences between some tissue types in terms of the identification of specific tissue structures. Thus, lungs are much more easily identified when bronchial cartilage and/or small deposits of anthracosis pigment are found, thereby also increasing the score rank of our evaluation scheme. Similarly, the identification of typical portal triads may facilitate the diagnosis “liver tissue.” Similarly, “key structures” are not present in other organs, such as kidney and the CNS. While the kidneys contain arteriolar blood vessels that might have even been identified even in less well-preserved status, the CNS proved to be the least well-preserved organ. Accordingly, in none of the cases we were able to identify CNS tissue in an adequately well-conservation status.

### 3.5. Identification of Pathologic Conditions

In our small series of cases some paleopathologic observations could be identified. This confirms the diagnostic power of paleohistology of soft tissues. Likewise, in one of the South American mummy skulls we detected an extensive bleeding within connective tissue structures obviously as a result of an acute rotational trauma [6]. One of the ancient Egyptian mummies revealed extensive intrapulmonary bleeding [7] most presumably due to an infection by a parasitosis. In another case, we were able to diagnose intramyocardial scarring of old-healed myocardial infarction [8]. The soft tissue sample from the rectal wall of a South American Inca mummy showed massive fibrosis of the submucosa, such as that seen in chronic inflammation due to Chagas-disease (*T. cruzi* infection) which was further confirmed in this particular case by molecular analysis [5]. Finally, the study of subcutaneous soft tissue samples from the Neolithic glacier mummy Ötzi allowed the diagnosis of an intravital laceration wound with bleeding residues with evidence for tissue reaction, such as that seen in shortly survived, but vital skin wounds [9]. Interestingly, even in relatively poorly conserved material some paleopathology was seen. In case 12, a crypt mummy with a postmortem time interval of 36 yrs. and the clinical diagnosis of metastatic tumor disease, an intrahepatic mass was seen highly suspicious for intrahepatic metastasis, despite the significant decomposition effects in the liver tissue.

## 4. Discussion

Despite its application even in early paleopathology at the beginning of the 20th century, soft tissue paleohistology has not been used widely. The reasons therefore may be multiple. First, paleohistology is a destructive technique and it is sometimes difficult (or even for ethical reasons impossible) to get access to the diagnostically relevant tissue/organ without major destruction of the mummy corpse; secondly, it requires rehydration, a “chemical” process that endangers the sample to be destroyed during the rehydration period; this issue may be different between mummified tissues from “cold” mummies (i.e., mummies that had kept frozen for a long period of time, but that obviously had retained a better hydration status than both natural and artificial mummies from “hot” desert areas); thirdly, there exists up to now no systematic study that provides information on the potential success rate of paleohistology. In contrast, major scientific information has as yet been obtained by paleohistology (e.g., [1–4]) and the observations of the present study confirm the potential of this technique in paleopathology.

We are aware that our present study has several limitations: (i) the number of investigated cases is low, but this is the as yet most extensive study on a systematic approach to paleohistology in terms of case numbers and time range, (ii) a certain preselection of macroscopically well-preserved material might have influenced the outcome, (iii) especially in most of the “older” cases (group II) we had access only to very limited tissues/organs.

Despite these restrictions we provide here interesting information on the tissue preservation in mummified corpses: (i) macroscopically well-preserved material has a considerably high rate of successful rehydration; (ii) “cold” mummies may be investigated with even higher success rates (this statement has to be made with great care since we have seen only one case!); (iii) there is no major difference between artificially and naturally mummified specimens (except when the artificial preservation is done by a typical fixative, such as ethanol (case “Wack-1”) or, since the end of the 19th century, formaldehyde); (iv) selected tissues/organs are better preserved than others (subcutis, blood vessels, lung versus CNS, and heart/skeletal muscle); (v) we present here an evaluation scheme that may serve as a basis for future systematic research.

In general, the evaluation scheme for the preservation of mummy tissue proved to be easily applicable and it showed surprisingly high values of reproducibility. However, we clearly admit that the scheme may be refined in ongoing studies, particularly when no prescreened mummies with macroscopic evidence for adequate conservation are used. Nevertheless, this study provides clear evidence that a paleohistologic study may be important in those cases with adequate macroscopic conservation and that those cases offer a considerably high rate of successive analysis.

As a further important observation, we detected that tissue preservation is less dependent from the postmortem time interval than from

- initial (i.e., within the first weeks/months) conservation of the mummy,
- type of tissue/organ under investigation (which in turn influences the diagnostic spectrum),
- experience in tissue preparation and histologic interpretation.

Accordingly, soft tissue pathology is more promising in those tissues with a high amount of connective tissue or “stable” tissue structures, such as fat tissue. These seem to undergo postmortem decomposition either very rapidly after death or remaining “stable” for a long period of time (proper storage provided). As the least well-conserved organ we identified the CNS; it is evident that CNS does not contain much connective tissue; furthermore, brain tissue is not characterized by specific substructures (with the exception of pigmented cells in the pigmented nuclei that may indeed be seen even in less well-preserved brain tissue specimens). In several instances, even paleohistology may not be decisive in identifying the proper organ. However, the identification of particular tissue structures and their conservation even after long periods of postmortem time interval seems to be a promising perspective for paleohistology.

Finally, recent significant progress in imaging techniques, mainly by CT scans, has opened the question whether paleohistology (as a destructive technique) should be applied to mummies at all. Problems in the proper identification of specific lesions and the need to distinguish between pre- and postmortem alterations, however, have made clear that CT scanning alone cannot solve distinct questions [17]. Additionally, the application of modern molecular techniques, such as stable isotope analysis or ancient DNA investigations, requires tissue material and can often be interpreted successfully when combined with corresponding paleohistology. Therefore, the careful, precise, and knowledgeable application of this analytical technique will persist to be fundamentally important for multidisciplinary mummy research.

## Figures and Tables

**Table 1:** Cases studied and results.

No.	ID	Origin	Age	Sex	Time	Skin	Conn	bl v	Fat t	Lung	Heart	Liver	Kidney	CNS
1	RM-1691	Germany	95	m	16 months	nd	3	3	3	2	2	nd	1	1
2	RM-1479	Germany	93	f	16 months	nd	3	3	3	2	1	nd	nd	1
3	RM-1689	Germany	79	f	17 months	nd	3	3	3	2	2	2	1	0
4	RM-1815	Germany	92	m	17 months	3	3	3	3	2	1	2	nd	1
5	RM-1480	Germany	81	f	17.5 months	nd	3	3	3	2	2	2	2	1
6	RM-1687	Germany	80	f	17.5 months	nd	2	3	3	nd	nd	2	nd	1
7	RM-1485	Germany	92	m	18 months	nd	3	3	2	2	1	nd	nd	nd
8	RM-1813	Germany	88	f	18 months	nd	3	3	3	2	2	2	2	1
9	RM-1811	Germany	82	f	18.5 months	nd	3	3	3	2	1	2	1	1
10	RM-1484	Germany	82	m	19 months	nd	3	3	3	2	2	2	2	0
11	RM-2418	Germany	78	f	5 yrs.	3	3	3	3	2	1	1	0	0
12	Path-12036	Germany	68	m	36 yrs.	2	3	3	3	2	2	1	nd	1
13	Wack-5	Germany	76	f	155 yrs.	2	3	3	3	2	1	1	1	0
14	Wack-4	Germany	88	m	163 yrs.	2	3	3	3	2	1	1	0	0
15	Wack-3	Germany	32	m	164 yrs.	2	3	3	3	2	2	1	1	1
16	Wack-2	Germany	69	m	173 yrs.	2	3	3	3	2	2	2	1	0

Gr. I	Mean value					2,3	2,9	3	2,9	2	1,5	1,6	1,1	0,6

	2SD					0,1	0,1	0	0,1	0	0,4	0,3	0,4	0,2

17	Wack-1^∗^	Germany	1	f	198 yrs.	4	4	4	4	4	4	4	4	3

18	BSAM	Peru?	20–25	f	370–560 yrs.	2	3	3	3	nd	nd	nd	nd	nd
19	LT-15-465	Peru	30–40	f	c. 1.650 yrs.	2	3	3	2	nd	nd	nd	nd	nd
20	CAH-331	Peru	60–70	m	c. 1.650 yrs.	3	3	2	2	nd	nd	nd	nd	1
21	CAH-427	Peru	20–30	f	c. 1.650 yrs.	2	2	2	3	nd	nd	nd	nd	1
22	TT-95-2	Egypt	50–60	f	c. 2.700 yrs.	2	3	3	2	nd	nd	nd	nd	nd
23	ÄS-12d	Egypt	20–40	m	c. 2.900 yrs.	2	2	2	2	2	1	2	nd	nd
24	DAN-95-21	Egypt	40–60	m	c. 2.900 yrs.	2	2	3	2	nd	2	nd	nd	nd
25	TT-95-22	Egypt	20–30	f	c. 3.000 yrs.	3	3	3	3	3	3	3	nd	nd
26	TT-183-4	Egypt	20–40	m	c. 3.200 yrs.	2	2	2	2	nd	nd	nd	nd	nd
27	TT-84-18	Egypt	20–30	m	c. 3.200 yrs.	2	3	2	2	2	nd	nd	1	nd
28	DAN-02-5	Egypt	20–30	f	c. 3.900 yrs.	nd	2	nd	nd	3	nd	nd	nd	nd
29	Ötzi	Alps	35–45	m	c. 5.200 yrs.	3	3	3	3	nd	nd	nd	nd	nd

Gr. II	Mean value					2,2	2,6	2,5	2,4	2,5	2	2,5	1	1

	2SD					0,1	0,3	0,2	0,2	0,4	0,5	0,5	0	0

nd: not determined.

^∗^Artificially mummified mummy; this case was not included in the statistical calculations of groups I and II.

**Table 2:** Evaluation criteria.

Points	Tissue overall structure (integrity)	Presence of specific (sub)structures	Presence of artefacts	Staining properties
0	Strong fragmentation, less than 25% of tissue intact	No substructures present/identifiable	Presence of numerous artefact foci (e.g., >10 crystal deposits >10 per HPF^∗^)	No specific staining (only nonspecific staining)
1	26–50% of tissue intact	Few substructures present (in 1–3 HPF^∗^)	Many artefact foci (5–10 artefacts per HPF^∗^)	Weak staining of specific structures
2	51–75% of tissue intact	Occasional substructures present (in 4–10 HPF^∗^)	Occasional artefact foci (1–4 artefact foci per HPF^∗^)	Moderate staining of specific structures
3	76–90% of tissue intact	Many substructures present (in 10–20 HPF^∗^)	No artefact foci	Good staining of specific structures
4	Tissue completely intact (91–100%)	Abundant substructures present (in >20 HPF^∗^)	—	—

^∗^HPF: high power field (visual field at magnification ×400).

**Table 3:** Scoring table.

Points	Score
0–3	0
4–6	1
7–9	2
10–12	3
13-14	4
